# Prognostic value of high EZH2 expression in patients with different types of cancer: a systematic review with meta-analysis

**DOI:** 10.18632/oncotarget.6612

**Published:** 2015-12-14

**Authors:** Tao Jiang, Yan Wang, Fei Zhou, Guanghui Gao, Shengxiang Ren, Caicun Zhou

**Affiliations:** ^1^ Department of Medical Oncology, Shanghai Pulmonary Hospital, Thoracic Cancer Institute, Tongji University School of Medicine, Shanghai, 200433, P.R. China

**Keywords:** enhancer of zeste homologue 2, cancer, prognosis, systematic review, meta-analysis

## Abstract

Enhancer of zeste homologue 2 (EZH2) is a potential independent mechanism for epigenetic silencing of tumor suppressor genes in cancer. We conducted an electronic search on PubMed, EMBASE, Web of Science, and Cochrane library to perform this up-to-date meta-analysis. Fifty-one studies with a total of 9444 patients were included. The prevalence of high EZH2 expression was 0.54 (95% CI: 0.47-0.61). High EZH2 expression was significantly associated with poorer prognosis [overall survival: HR 1.54 (95% CI: 1.30-1.78), *P* < 0.000; disease free survival: HR 1.35 (95% CI: 1.00-1.71), *P* < 0.000]. In breast cancer, high EZH2 expression correlated with histological types [OR: 1.53 (95CI: 1.13-2.06); *P* < 0.006], histological grade [OR: 1.62 (95CI: 1.35-1.95); *P* < 0.000], estrogen receptor (ER) negativity [OR: 2.05 (95CI: 1.67-2.52); *P* < 0.000], progesterone receptor (PgR) negativity [OR: 1.42 (95CI: 1.03-1.96); *P* = 0.034], HER-2 positivity [OR: 1.35 (95CI: 1.08-1.69); *P* = 0.009], and high p53 expression [OR: 1.66 (95CI: 1.07-2.59); *P* = 0.024]. These results suggest that high EZH2 expression may be a promising prognostic factor to different cancers. High EZH2 expression tends to correlate with pathological types, histological grade, ER negativity, PgR negativity, HER-2 positivity and p53 high expression in breast cancer.

## INTRODUCTION

Enhancer of zeste homologue 2 (EZH2) is a catalytic subunit of the polycomb repressive complex 2 (PRC2), which represses gene expression by methylating lysine 27 of histone 3 (H3K27)[1]. EZH2-mediated methylation plays a pivotal role in epigenetic silencing of tumor suppressor genes in cancer and is involved in fundamental cellular processes, such as cell fate decision, cell cycle regulation, senescence and cell differentiation [2–6]. Hyperactivation of EZH2, either by high expression or mutations, is found in a variety of malignancies including breast, prostate, lung, gastric, and renal cell cancers in addition to glioblastoma[7–9].

EZH2 is highly expressed in a wide range of cancer types. In cell lines, enforced expression of EZH2 could increase proliferation and oncogenic capacity. Overexpressing EZH2 in mammary epithelial cells of the tumorigenic mouse model using mammary tumor virus long terminal repeat (MMTV-EZH2) leads to epithelial hyperplasia phenotype[10]. Previous studies have showed that high expression of EZH2 was correlated with aggressiveness, metastasis, and poor prognosis in breast, prostate, bladder and renal cell cancer[11–13]. Recently, some studies also demonstrated that high EZH2 expression was also associated with poor prognosis in lung and gastric cancer[14, 15], glioblatoma, head and neck squamous cell carcinoma[8, 16]. However, there are still quite a number of studies reported that there is no correlation between high EZH2 expression and prognosis in cancers mentioned above[17–19]. Moreover, some studies showed that high expression of EZH2 was the better prognostic factor in non-small-cell lung cancer (NSCLC) and colorectal cancer (CRC)[20, 21]. Although there have been already a meta-analysis on this issue, several important articles are not included in that paper and the subgroup analysis give little useful information[22]. Hence we performed this up-to-date systematic review with meta-analysis on the influence of high EZH2 expression on the outcomes of different cancers, as well as the incidence of high EZH2 expression, and to provide an overview of the current status of high EZH2 expression in tumor progression and therapy.

## RESULTS

### Study selection

**Figure 1:**
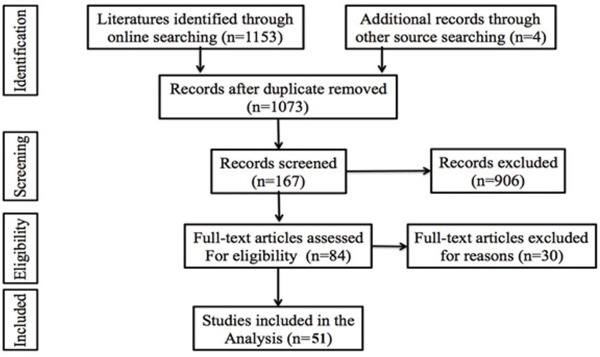
Flow diagram of the study selection process

The result of literature inclusion was showed in Figure 1. A total of 1073 potentially relevant articles were found, and 51 studies were included in this analysis after screening[4, 6, 8, 11–21, 23–59]. Most of the excluded abstracts were reviews or studies with insufficient data.

### Characteristics of the studies

In this analysis, 9444 cases from 51 studies were used to study high EZH2 expression in 18 kinds of human tumors. Sixteen of 51 studies were in breast cancer, 10 studies were in lung cancer, 7 studies were in CRC and other 21 studies were about digestive, gynecological and urinary cancers. The main characteristics of the included studies were shown in Table 1. In addition prognostic data were obtained from 16 of 54 studies on disease free survival (DFS) and 43 of 54 studies on overall survival (OS).

**Table 1:** Characteristics of included studies

Cancer Types	Author	Year	Patients' Number	Rate of High EZH2 Expression	Test Method	Definition of High Expression
Breast cancer	Kleer	2003	280	40.71%	IHC+qRT-PCR	3-4 score ( 4-value intensity score) defined high expression
	Collett	2006	190	47.37%	IHC	staining index (values 0-9) >3*
	Pietersen	2008	295	40.34%	IHC	NC
	Athanassiadou	2011	100	64.00%	IHC	>10% of nuclear staining
	Gong	2011	88	63.64%	IHC	>10% of nuclear staining
	Reijm	2011	278	66.55%	IHC+qRT-PCR	NC
	Alford	2012	480	42.50%	IHC	>25% of nuclei staining
	Brot	2012	140	85.71%	IHC	>25% of nuclei staining
	Hussein	2012	261	33.33%	IHC	nuclear staining was scored based on intensity (0-3) and ≥2 defined high expression
	Jene-Sanz	2013	95	43.16%	IHC+qRT-PCR	NC
	Knudsen	2013	236	71.61%	IHC	>15% of nuclear staining
	Panousis	2013	105	57.14%	IHC	>10% of nuclei staining
	Roh	2013	49	67.35%	IHC	>25% of nuclei staining
	Bae	2014	146	49.32%	IHC	>30% of nucleai staining
	Dong	2014	410	24.15%	IHC	staining index (values 0-9) >3*
	Reijm	2014	250	46.40%	IHC	1
Lung cancer	Kikuchi	2010	154	62.34%	IHC	>25% of nuclei staining
	Takawa	2011	292	46.23%	IHC+qRT-PCR	NC
	Cao	2012	94	62.77%	RT-PCR	A fold difference >1 is considered high EZH2 expression
	Huqun	2012	106	62.26%	IHC	>50% of nuclei stained
	Lv	2012	69	63.77%	IHC+qRT-PCR	>25% of nuclei staining
	Behrens	2013	541	26.80%	IHC	final score (values 0-300) >125**
	Chen	2013	42	42.86%	IHC	final IHC score (values 0-12) >3***
	Wan	2013	113	50.44%	IHC	NC
	Xu	2014	360	56.67%	IHC	staining index (values 0-9) >3*
	Geng	2015	195	49.23%	IHC	≥30% of tumor cells with strong staining intensity=2
Colorectal cancer	Mimori	2005	61	52.46%	qRT-PCR	> the median tumor/normal ratio
	Fluge	2009	241	17.01%	IHC	staining index (values 0-9) >3*
	Wang	2010	119	69.75%	IHC	>30% of nucleai staining
	Takawa	2011	172	91.86%	IHC	NC
	Benard	2014	247	23.89%	IHC+qRT-PCR	> median percentage of positively stained nuclei in the marked area
	Meng	2014	112	52.68%	IHC	>50% of nucleai staining
	Liu	2015	82	80.49%	qRT-PCR	> the median tumour/normal ratio
Bladder cancer	Raman	2005	24	87.50%	IHC	staining index (values 0-9) >3*
	Hinz	2008	99	90.91%	qRT-PCR	> median expression levels
Prostate cancer	Bachmann	2006	104	8.65%	IHC	staining index (values 0-9) >3*
	Li	2011	129	44.96%	IHC	staining index (values 0-9) >3*
Renal cancer	Hinz	2013	101	56.44%	IHC+qRT-PCR	> median expression levels
	Wagener	2009	411	11.19%	IHC	>25% of nucleai staining
	Liu	2010	373	52.82%	IHC	final IHC score (values 0-12) ≥2***
Glioblastoma	Wu	2013	128	62.50%	IHC	staining index (values 0-9) >4.5*
	Zhang	2013	83	51.81%	IHC	≥2 (scored 0 to 3) indicated high expression
Digestive cancer	He	2015	98	54.08%	IHC	>50% of nucleai staining
	Liu	2010	108	53.70%	IHC	>25% of nuclei staining
	Ha	2011	164	52.44%	IHC	final IHC score (values 0-12) ≥10***
	He	2012	117	70.09%	IHC	>50% of nucleai staining
	Lee	2012	178	92.13%	IHC	staining index (values 0-9) >median score*
	Li	2012	84	64.29%	IHC	final IHC score (values 0-12) ≥4***
	Zhang	2013	167	76.05%	IHC	staining index (values 0-9) >3*
Gynecological cancer	Bachmann	2006	276	17.75%	IHC	staining index (values 0-9) >3*
	Rao	2010	179	49.72%	IHC	>50% of nucleai staining
	Liu	2014	101	68.32%	IHC	staining index (values 0-9) ≥4*
Head and neck cancer	Cao	2014	117	50.43%	IHC	cutoff value was set as the median of the labeling index

* staining index (values 0-9) = staining intensity (0-3) × proportion of immunopositive cells (<10% = 1, 10-50% = 2, >50% = 3).

** The final score was then obtained by multiplying the intensity and extension values (range, 0-300) by using a 4-value intensity score (0, 1, 2, and 3) and the percentage (0-100%) of the extent of reactivity in each core.

*** the number of positive cancerous cells was estimated as follows (0, no positive cells; 1, 0-25% positive cells; 2, 25-50% positive cells; 3, 50-75% positive cells; and 4, 75-100% positive cells). These scores were multiplied with an intensity scale (0, negative; 1, weak; 2, moderate; and 3, intensive), and the final score ranged from 0-12; NC: not clear.

### Method of evaluation High EZH2 expression

Quantitative reverse transcription-polymerase chain reaction (qRT-PCR) and immunohistochemistry (IHC) staining were used to test high EZH2 expression. IHC was the most commonly used method (47 of 54) including 7 of them using both qRT-PCR and IHC. It is noteworthy that the criteria for high EZH2 expression were highly heterogeneous among different studies using IHC. For example, in some studies, the percentage of positive-staining tumor cells larger than 10%, 25%, 30% or 50% were considered to be high EZH2 expression. In other studies, staining intensity > 50% was taken as high EZH2 expression. Immunoreactivity scores (IRS) was commonly used criterion, which were obtained for each case by multiplying the percentage and intensity score. The percentage scoring of immunoreactive tumor cells was as follows: 0 (0%), 1 (1-10%), 2 (11-50%) and 3 (> 50%). The staining intensity was visually scored and stratified as follows: 0 (negative), 1 (weak), 2 (moderate) and 3 (strong). However, in these studies used IRS, the cutoff values were different among different studies.

### Prevalence of high EZH2 expression

**Figure 2:**
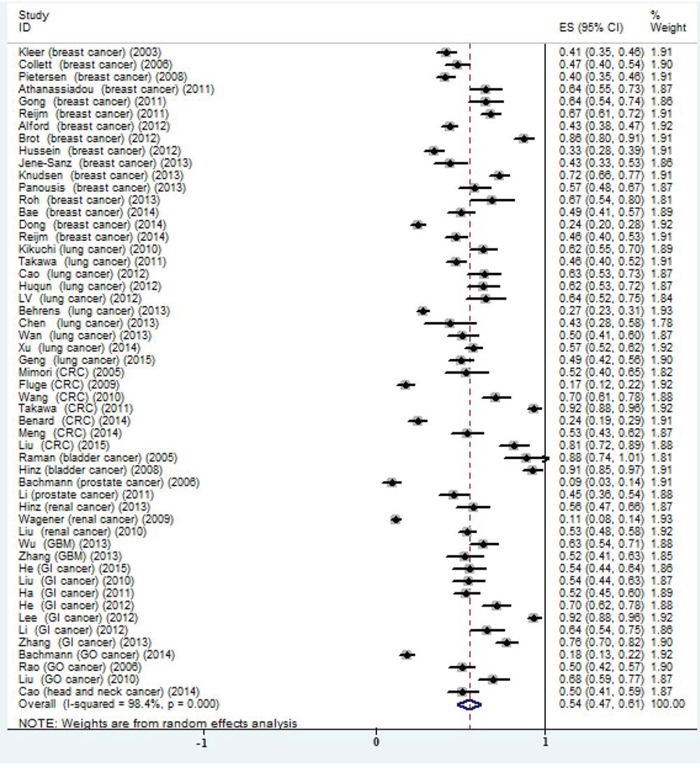
Meta-analysis of the prevalence of EZH2 high expression in all included studies

The prevalence of high EZH2 expression in these studies ranged from 8.65% to 92.13%, partly reflecting the heterogeneity in the criteria for high expression. In the meta-analysis of 51 studies, the prevalence of high EZH2 expression was 0.54 (95% CI: 0.47-0.61) and large heterogeneity existed (I^2^ = 98.4%; *P* = 0.000; Figure 2). Subgroup analysis was stratified by test methods and evaluation criteria, but the heterogeneity could not be reduced (Supplementary Figures S1-S2). In subgroup analysis, the rates of high EZH2 expression in breast cancer, lung cancer and CRC were 0.53 (95%CI: 0.44-0.62), 0.52 (95%CI: 0.42-0.62) and 0.55 (95%CI: 0.29-0.82), respectively.

### Meta-analysis of high EZH2 expression and cancer prognosis

**Figure 3:**
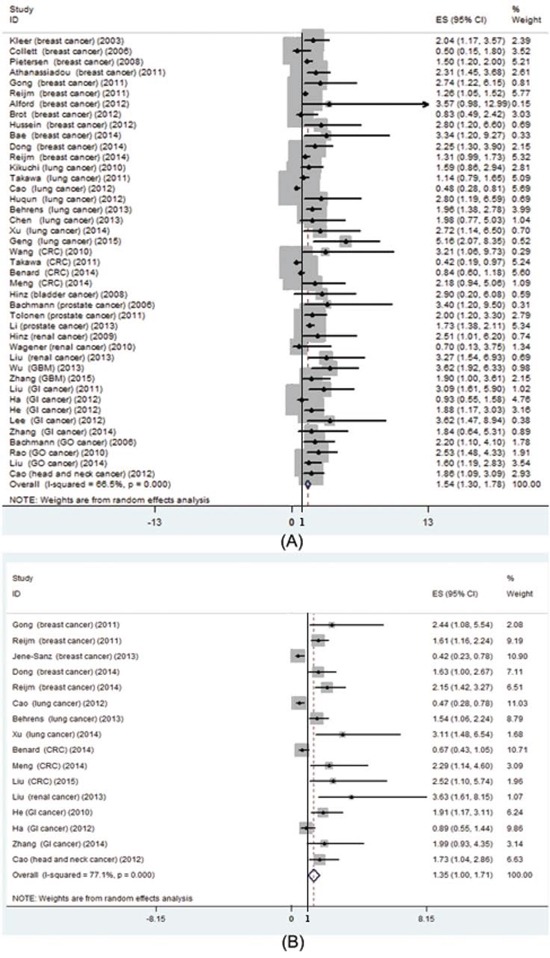
Prognostic value of EZH2 high expression in patients with cancer **A.** meta-analysis of EZH2 high expression and overall survival in various cancers; **B.** meta-analysis of EZH2 high expression and disease free survival in various cancers.

Pooled OS was used to illustrate high EZH2 expression overall effect for the studies containing prognostic data. Meta-analysis of high EZH2 expression status and OS in a variety of cancers was performed; 8252 patients in 43 studies for high EZH2 expression were included. The results showed that the pooled HRs were significant for high EZH2 expression [HR 1.54 (95% CI: 1.30-1.78); *P* = 0.000] (Figure 3A). Pooled HRs > 1 indicated that high EZH2 expression would be correlated with poor OS in various cancers. However, the results showed high heterogeneity (I^2^ = 66.5%; *P* = 0.000). In the sixteen studies that reported DFS, the pooled result indicated that high EZH2 expression was also related to shorter DFS [HR: 1.35 (95% CI: 1.0-1.71); *P* = 0.000; Figure 3B]. The results also showed high heterogeneity (I^2^ = 77.1%; *P* = 0.000).

**Figure 4:**
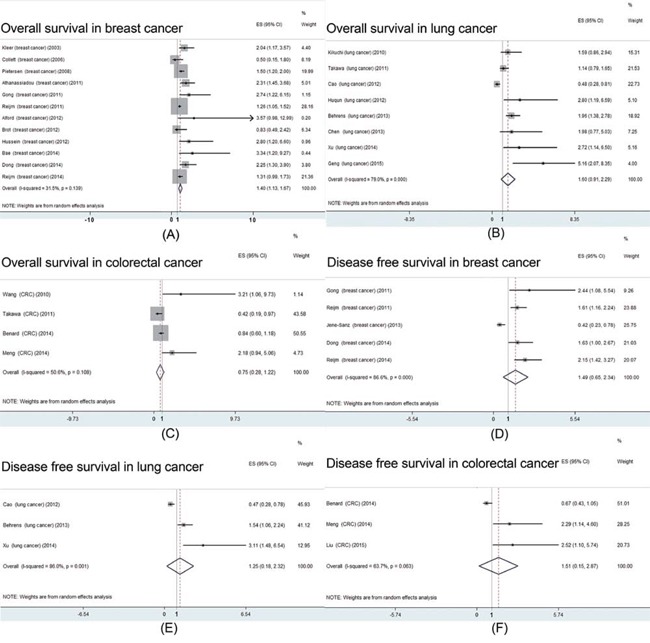
Prognostic value of EZH2 high expression in patients with breast, lung or colorectal cancer **A.** meta-analysis of EZH2 high expression and overall survival in patients with breast cancer; **B.** meta-analysis of EZH2 high expression and overall survival in patients with lung cancer; **C.** meta-analysis of EZH2 high expression and overall survival in patients with colorectal cancer; **D.** meta-analysis of EZH2 high expression and disease free survival in patients with breast cancer; **E.** meta-analysis of EZH2 high expression and disease free survival in patients with lung cancer; **F.** meta-analysis of EZH2 high expression and disease free survival in patients with colorectal cancer;

In addition we carried out the meta-analysis of high EZH2 expression and prognosis in breast, lung and colorectal cancer, respectively. The results showed that high EZH2 expression was correlated with poor OS [HR: 1.40 (95% CI: 1.13-1.67); *P* = 0.000; Figure 4A] in breast cancer instead of lung [HR: 1.60 (95% CI: 0.91-2.29); *P* = 0.376; Figure 4B] and CRC [HR: 0.75 (95% CI: 0.28-1.22); *P* = 0.340; Figure 4C]. All three pooled results indicated that high EZH2 expression was not related to shorter or longer DFS, respectively (Figure 4D–4F).

### High EZH2 expression and clinicopathological features

To explore the relationship between high EZH2 expression and clinicopathological parameters, subgroup analyses were performed according to the different cancer types. The details were summarized in Table 2. As the results suggested, in breast cancer, high EZH2 expression was associated with pathological types [OR: 1.53 (95CI: 1.13-2.06); *P* = 0.006], histological grade [OR: 1.62 (95CI: 1.35-1.95); *P* = 0.000], estrogen receptor (ER) negativity [OR: 2.05 (95CI: 1.67-2.52); *P* = 0.000], progesterone receptor (PgR) negativity [OR: 1.42 (95CI: 1.03-1.96); *P* = 0.034], HER-2 positivity [OR: 1.35 (95CI: 1.08-1.69); *P* = 0.009], and p53 high expression [OR: 1.66 (95CI: 1.07-2.59); *P* = 0.024]. In CRC and lung cancer, high EZH2 expression was not correlated with the reported clinicopathological features (Table 2).

**Table 2:** The association between clinicopathological parameters and EZH2 high expression with respect to patients with different cancers

Factors	No. of study	OR (95%CI)	p value	Heterogeneity	
				I2	p value
Breast Cancer					
Age >65/<65	4	0.917 (0.721-1.167)	0.483	0.0%	0.586
Ethnicity (Asian/Caucasian)	16	1.133 (0.632-2.030)	0.762	76.6%	0.039
Histology (ductal/other)	7	1.526 (1.130-2.062)	0.006	0.0%	0.773
Histological grade (III/I-II)	9	1.620 (1.349-1.947)	0.000	46.8%	0.059
Lymph node status (P/N)	9	1.106 (0.876-1.397)	0.397	32.3%	0.160
ER status (N/P)	8	2.053 (1.673-2.521)	0.000	3.10%	0.406
PR status (N/P)	8	1.420 (1.027-1.962)	0.034	54.8%	0.030
HER-2 (P/N)	10	1.348 (1.078-1.685)	0.009	0.0%	0.792
p53 (H/L)	4	1.664 (1.069-2.588)	0.024	35.5%	0.199
Lung Cancer					
Age (>65/<65)	5	1.014 (0.817-1.258)	0.901	0.0%	0.941
Gender (male/female)	7	1.041 (0.768-1.412)	0.793	52.5%	0.049
Ethnicity (Asian/Caucasian)	10	1.523 (0.932-2.474)	0.313	50.4%	0.049
Smoking (non/smoker)	5	0.940 (0.551-1.663)	0.831	75.4%	0.003
Histology (Ade/other)	5	1.099 (0.639-1.890)	0.733	78.2%	0.001
Differentiation (well/poor)	6	0.749 (0.445-1.260)	0.276	66.7%	0.010
Lymph node (N/P)	3	0.816 (0.411-1.620)	0.561	86.4%	0.001
Stage (I/II-IV)	4	0.888 (0.563-1.402)	0.611	52.9%	0.095
Colorectal Cancer					
Gender (male/female)	3	0.970 (0.715-1.316)	0.845	0.0%	0.767
Differentiation (well/poor)	3	0.806 (0.515-1.262)	0.348	0.0%	0.690
Lymph node (N/P)	4	0.890 (0.666-1.189)	0.432	0.0%	0.622

Abbreviations: No.: number; OR: odds ratio; CI: confidence interval; Ade: adenocarcinoma; P: positive; N: negative; H: high expression; L: low expression

### Sensitivity and publication bias

**Figure 5:**
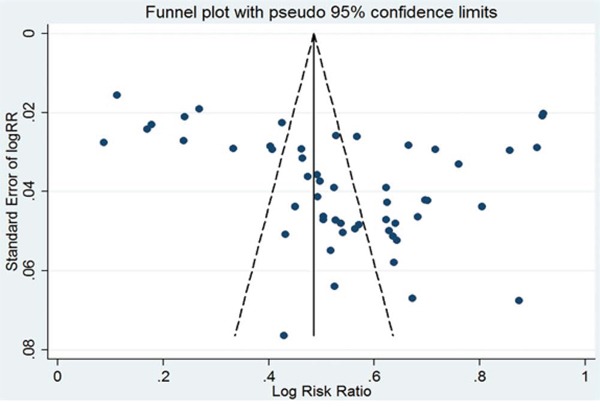
Publication bias for the prevalence of EZH2 high expression in various cancers

Analysis of sensitivity was performed by omitting one study at one time to measure its effect on prevalence and pooled HRs. Deletion of the study by Li et al. [56] significantly reduced the heterogeneity in the analysis of high EZH2 expression and OS. No other individual study influenced the results. Begg's funnel plots and Egger's tests evaluated the publication bias, and it was only detected in the analysis of high EZH2 expression prevalence (*P* = 0.021 for Egger's test). In the other analyses, the Begg's funnel plots were almost symmetric (Figure 5) and Egger's tests indicated that there was no evidence of publication bias.

## DISCUSSION

In this article, the results indicated that EZH2 highly expressed in 18 kinds of human cancers and the incidence of high EZH2 expression was 0.54 (95% CI: 0.47-0.61). More importantly, the results of all included studies demonstrated that high expression of EZH2 could be the prognostic factor for both OS and DFS [HR: 1.54 (95% CI: 1.30-1.78), HR: 1.35 (95% CI: 1.0-1.71); *p* = 0.000; respectively]. In virtue of the high heterogeneity, we performed the subgroup analysis on breast, lung and colorectal cancer. The results showed that high EZH2 expression was associated with poor OS in breast cancer instead of lung and CRC. All three pooled results indicated that high EZH2 expression was not related to shorter or longer DFS. In breast cancer, EZH2 inhibited the tumor suppressor RKIP transcription through repression-associated histone modifications, therefore promoting tumor progression and metastasis[5]. This may partly explain the reason why high EZH2 expression was correlated with poor OS in breast cancer. As to the clinicopathological parameters, we found that high EZH2 expression was associated with ER negativity, PgR negativity, HER-2 positivity and p53 high expression. These results are concordant with previous study. In addition, we also found an association between high EZH2 expression and histological grade. This would also be part of reason that high EZH2 expression was correlated with poor OS in breast cancer.

Another important issue is the assessment of high EZH2 expression. To date, there is no golden standard for scoring EZH2 expression. Our study collected all test methods and scoring system from the included studies (Table 1). The method reported by Bachmann *et al.* [12] was widely used (10 studies). They defined EZH2 positive expression via the product of intensity and quantity (cutoff value > 3). However, other studies used different criteria including 3 studies used the nuclear immunostaining > 10%, 7 studies used > 25%, 3 studies used > 30%, 5 studies used > 50% and 4 studies used the final IHC score ≥4. This would be the major reason on high heterogeneity of high EZH2 expression rate. Recently, the researcher used a sum of intensity and quantity with a cutoff value of >2 to quantify tumors as high EZH2 expression and obtained a similar association between EZH2 protein expression and progress free survival (PFS)[35]. Thus, the relation seems to be independent of the method of scoring, as long as the amount proportion of stained cells is included since intensity by itself did not associate with PFS, whereas quantity did according to different methods.

Accumulated evidence indicates that EZH2 contributes to various aspects of cancer by regulating a variety of target genes. It was shown that EZH2-containing PRC2 transcriptionally inhibited cell cycle suppressor INK-ARF to drive cell cycle progression, prevent cell senescence and also exhaustion of cancer stem cells. In addition, EZH2 promotes epithelial-mesenchymal transition (EMT), a process that is associated with cancer progression and metastasis, by interacting with transcription factor SNAIL1 and suppressing expression of epithelial marker E-cadherin (CDH1). Moreover, EZH2 is implicated in promoting tumor angiogenesis. It shows that vascular endothelial growth factor (VEGF), which stimulates angiogenesis, can increase E2F1/3 transcription factors to transactivate EZH2 expression. Increased EZH2 expression by VEGF silences expression of a negative regulator of angiogenesis, vasohibin1 (VASH1), and subsequently enhances angiogenesis. Another recent study further identified that under hypoxia insult, induced EZH2 expression decreases DNA damage repair protein RAD51 expression, which leads to genomic aberrations, such as RAF1 gene amplification, to promote RAF1-ERK-β-catenin signaling and expansion of breast tumor initiating cells. Taken together, EZH2 plays an essential and multi-faceted role in human cancers. Blocking EZH2 expression or activity may represent a promising strategy for anti-cancer treatment targeting tumor cells, tumor endothelial cells and tumor stem cells.

To date, several potent inhibitors of EZH2 have been discovered. One of the most valuable inhibitors is 3-deazaneplanocin A (DZNep). DZNep is a *S*-adenosylhomocysteine hydrolase inhibitor. It can deplete EZH2 and the associated H3K27me3 and induce apoptosis in breast and colon cancer cells[60]. More importantly, at the animal level, DZNep could affect the epigenetic pathway with minimal toxicity. Similarly, DZNep was shown to be effective in inhibiting prostate cancer cell growth and its anti-tumor activity is in part mediated by suppressing the tumorigenic potential of the prostate cancer stem cell[61]. However, all of the reported inhibitors were just confirmed effective in cell lines and/or mice based experiments. The therapeutic value of these inhibitors needs to be further assessed.

When interpreted the results, some limitations of our meta-analysis should be acknowledged. Firstly, it is possible that there may be some degree of publication bias in this area of research. We identified several abstracts describing articles that were not further detailed in standard publications. We have made any effort to contact authors of primary studies. However, we have not received any reply. Hence, we could not include these articles in the review. Secondly, there was statistical heterogeneity among the studies regarding the prevalence of high EZH2 expression. Studies may have differed with regard to the baseline characteristics of the patients included age, histological type, differentiation, disease stage, the duration of follow-up and adjustments for other cofactors. Fortunately, we found that the heterogeneity may be due to the lack of test method and evaluation criteria. Thirdly, there is clearly a multitude of confounding factors (laboratory condition, test technique and so on) that make experiment comparisons difficult.

In conclusion, the current evidence suggests that high EZH2 expression may be a promising prognostic factor to human cancers, especially the breast cancer. High expression of EZH2 trends to correlate with histological grade, ER negativity, PgR negativity, HER-2 positivity and p53 high expression. EZH2 is an attractive target in future cancer treatment. In addition, some questions should be illustrated clearly, including the test method and evaluation criteria of high EZH2 expression before large scale clinical studies being conducted.

## METHODS

### Publication search strategy

We performed a comprehensive publication search through the PubMed, EMBASE, Web of Science, and Cochrane library up to March 31, 2015, without language limitations. The following contextual query language was used: (“Enhancer of Zeste Homologue 2” OR “EZH2”) AND (“cancer” OR “carcinoma” OR “neoplasm”). Titles and abstracts were reviewed to identify reports, which examined the association of EZH2 expression with clinical outcomes, such as overall survival (OS), disease free survival (DFS) and clinicopathological features. Reference lists of identified studies and reviews were also handsearched. We have made any effort to contact authors of primary studies. This analysis was performed in accordance with Preferred Reporting Items for Systematic Reviews and Meta-Analyses: the PRISMA Statement[62].

### Study selection

The criteria for inclusion were listed as follows: (1) studied high EZH2 expression in any type of human cancers; (2) the expression of EZH2 was detected on tumor tissue, rather than in the serum or cell lines or any other kinds of specimens; and (3) reported data necessary to calculate the incidence of high EZH2 expression and/or high risk (HR) on survival outcomes. Studies were excluded if they were: (1) reviews, case-only studies, or familial studies; (2) lacking sufficient data for calculation of incidence and/or HR with 95% confidence intervals (CIs); and (3) duplication of previous publications or replicated samples. Two reviewers determined study eligibility independently. Disagreements were solved by consensus.

### Data extraction

From each study, the following information was extracted: first author's name, year of publication, study population, EZH2 expression assessment methods, cut-off definition, and incidence of high EZH2 expression with 95% CIs, HR for OS, and/or DFS with corresponding 95% CIs. If the HRs and CIs were not reported, the total observed death events and the numbers of patients in each group were extracted to calculate HR and its variance indirectly. In order to guarantee the accuracy of collected data, studies for which only Kaplan-Meier curves would not be included. When both univariate analysis and multivariate analysis were reported to get the HR, the results of multivariate analysis were selected. Two reviewers extracted the data independently, using a predefined Excel form. Disagreements were resolved by consensus after discussion. If they can't get the consensus, the article would be excluded.

### Quality assessment

Two reviewers assessed the study quality independently by using the following factors: (1) distinct definition of the study population and the type of cancer; (2) clear definition of the test method and the cut-off value of high EZH2 expression; (3) sample size larger than ten and (4) clear definition of the outcome assessment (if applicable). Studies lacking any of these criteria were excluded.

### Statistical analysis

For the incidence of high EZH2 expression, the incidences and 95% CIs were combined. Dichotomous data were compared using an odds ratio (OR). Respective 95% CIs were calculated for each estimate and presented in forest plots. For time-to-event data, the HRs with their 95% CIs were directly extracted from the research article or calculated using previously published methods proposed by Tierney et al. [63]. The χ^2^ test was used to test for statistical heterogeneity and the I^2^ statistic was used to assess the extent of variability attributable to statistical heterogeneity across trials. P > 0.1 for the χ^2^ test and I^2^ < 25% were interpreted as signifying low-level heterogeneity. When there was no statistically significant heterogeneity, a pooled effect was calculated with a fixed-effects model; otherwise, a random-effects model was used.

To investigate the source of heterogeneity, predefined subgroup analyses were performed based on cancer type and assessment method. Sensitivity analyses were performed to assess the stability of the results, namely, a single study was deleted each time to reflect the influence of the individual data set on the results. Begg's funnel plots and Egger's tests were used to assess publication bias. Furthermore, we explored performed subgroup analysis on the relationship between high EZH2 expression and clinicopathological parameters in breast, lung and colorectal cancer.

Statistical analysis was performed by STATA v12.0 (Stata Corporation, TX) and Review Manager 5.0 software. All data were analyzed using the Statistical Package for Social Sciences (SPSS) software (version 16.0 for Windows). *P* < 0.05 was considered statistically significant except for the Q-test.

## SUPPLEMENTARY FIGURES


