# Supplementary material for: Prognostic value of high EZH2 expression in patients with different types of cancer: a systematic review with meta-analysis
Source: Oncotarget. 2015 Dec 14;7(4):4584–97. doi: 10.18632/oncotarget.6612 (PMC4826228; doi:10.18632/oncotarget.6612)
Supplement: Supplementary file 1 [file oncotarget-07-4584-s001.pdf]

SUPPLEMENTARY FIGURES

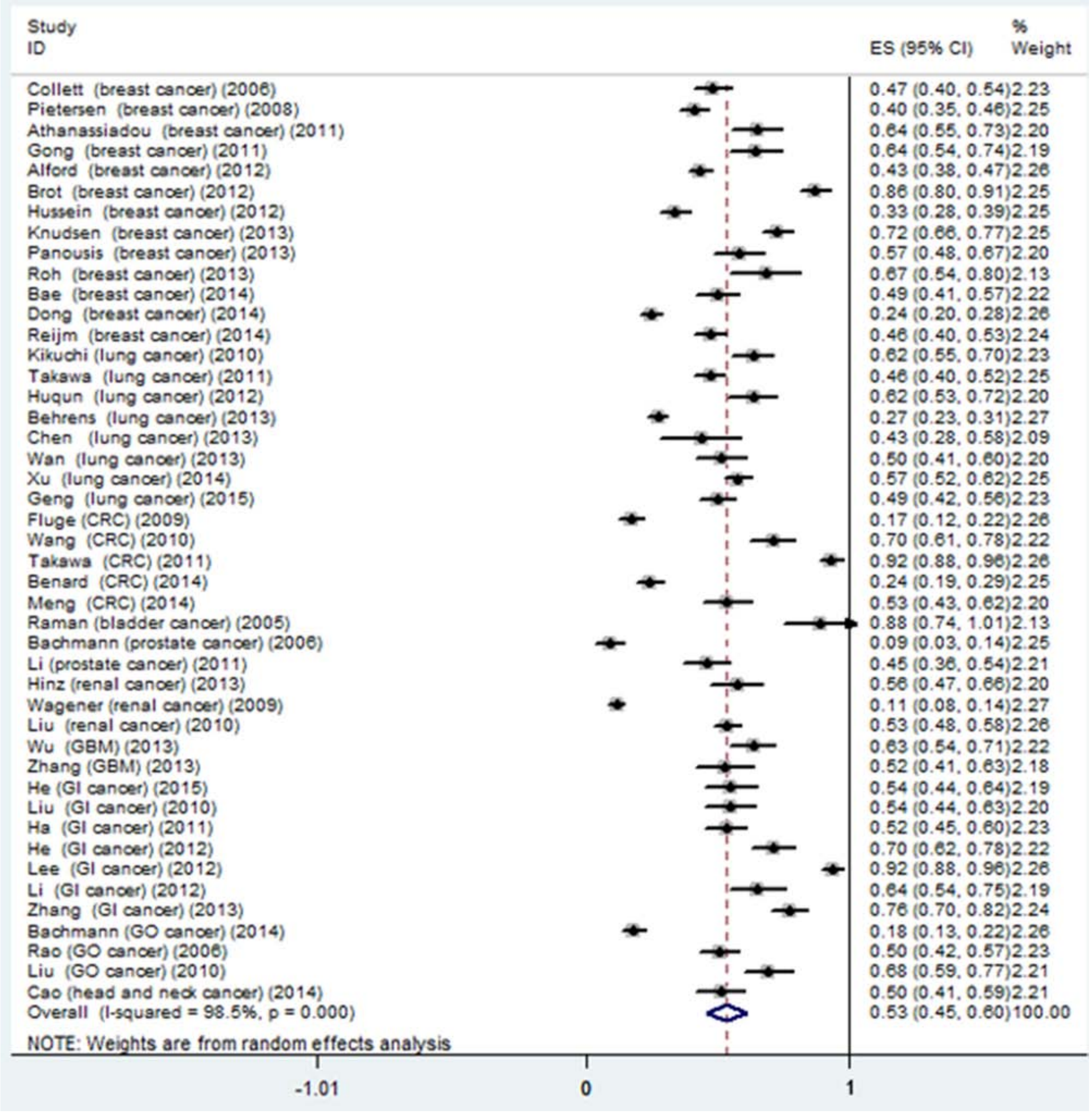

Supplementary Figure S1: Meta-analysis of the prevalence of EZH2 high expression by same test method.

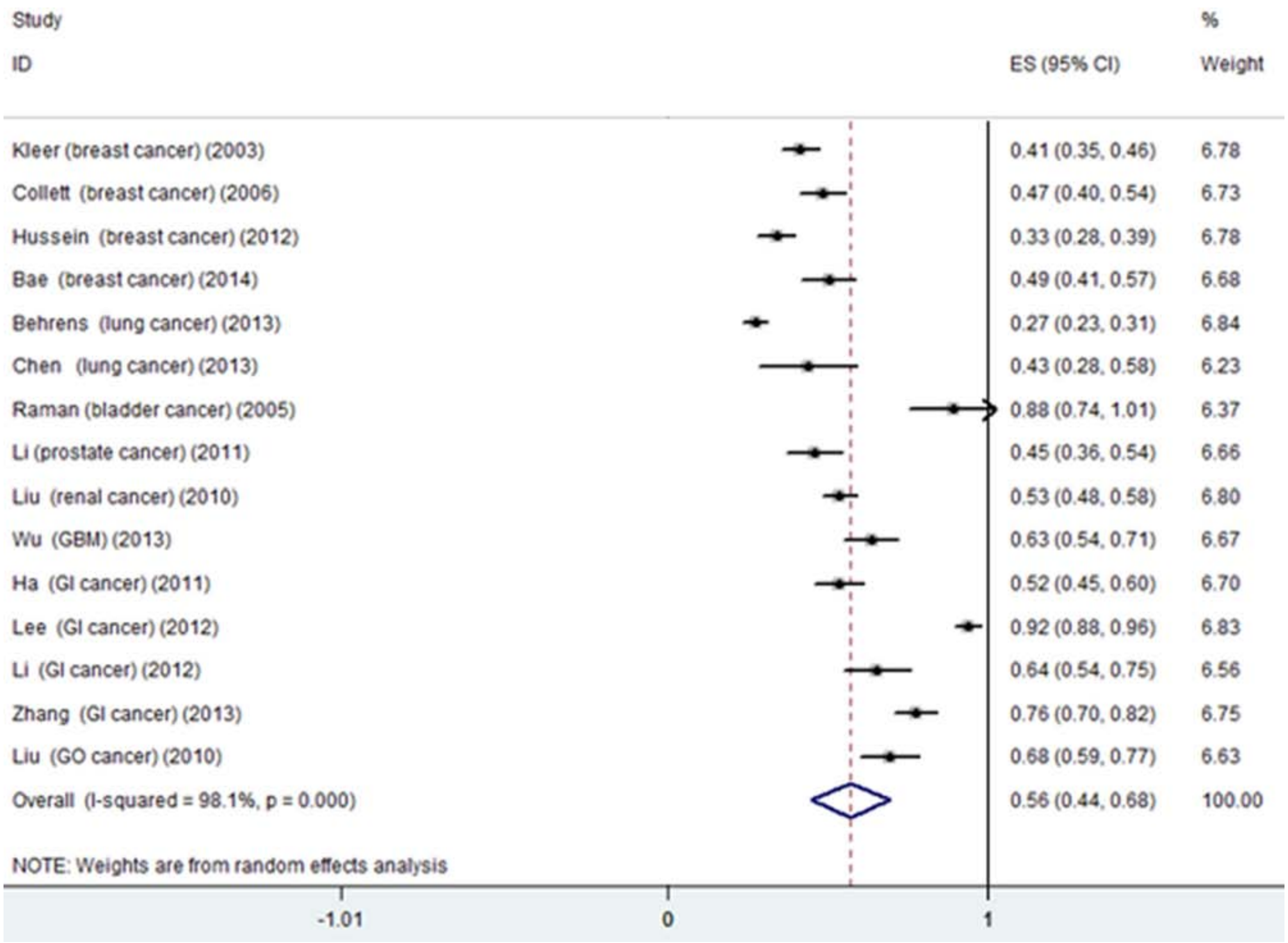

Supplementary Figure S2: Meta-analysis of the prevalence of EZH2 high expression by same definition of EZH2 high expression.
